# A General Safety Assessment for Purified Food Ingredients Derived From Biotechnology Crops: Case Study of Brazilian Sugar and Beverages Produced From Insect-Protected Sugarcane

**DOI:** 10.3389/fbioe.2018.00045

**Published:** 2018-04-26

**Authors:** Reese D. Kennedy, Adriana Cheavegatti-Gianotto, Wladecir S. de Oliveira, Ronald P. Lirette, Jerry J. Hjelle

**Affiliations:** ^1^Hjelle Advisors LLC, Clayton, MI, United States; ^2^Regulatory Department, Centro de Tecnologia Canavieira, Piracicaba, Brazil; ^3^Ron Lirette Biotech Consulting LLC, Theriot, LA, United States

**Keywords:** sugar, highly purified substances, Cry1Ab, Cry1Ac, NPTII, bar, *Saccharum*, sugarcane

## Abstract

Insect-protected sugarcane that expresses Cry1Ab has been developed in Brazil. Analysis of trade information has shown that effectively all the sugarcane-derived Brazilian exports are raw or refined sugar and ethanol. The fact that raw and refined sugar are highly purified food ingredients, with no detectable transgenic protein, provides an interesting case study of a generalized safety assessment approach. In this study, both the theoretical protein intakes and safety assessments of Cry1Ab, Cry1Ac, NPTII, and Bar proteins used in insect-protected biotechnology crops were examined. The potential consumption of these proteins was examined using local market research data of average added sugar intakes in eight diverse and representative Brazilian raw and refined sugar export markets (Brazil, Canada, China, Indonesia, India, Japan, Russia, and the USA). The average sugar intakes, which ranged from 5.1 g of added sugar/person/day (India) to 126 g sugar/p/day (USA) were used to calculated possible human exposure. The theoretical protein intake estimates were carried out in the “Worst-case” scenario, assumed that 1 μg of newly-expressed protein is detected/g of raw or refined sugar; and the “Reasonable-case” scenario assumed 1 ng protein/g sugar. The “Worst-case” scenario was based on results of detailed studies of sugarcane processing in Brazil that showed that refined sugar contains less than 1 μg of total plant protein /g refined sugar. The “Reasonable-case” scenario was based on assumption that the expression levels in stalk of newly-expressed proteins were less than 0.1% of total stalk protein. Using these calculated protein intake values from the consumption of sugar, along with the accepted NOAEL levels of the four representative proteins we concluded that safety margins for the “Worst-case” scenario ranged from 6.9 × 10^5^ to 5.9 × 10^7^ and for the “Reasonable-case” scenario ranged from 6.9 × 10^8^ to 5.9 × 10^10^. These safety margins are very high due to the extremely low possible exposures and the high NOAELs for these non-toxic proteins. This generalized approach to the safety assessment of highly purified food ingredients like sugar illustrates that sugar processed from Brazilian GM varieties are safe for consumption in representative markets globally.

## Introduction

In Brazil alone, sugarcane borer, a lepidoptera that feeds on sugar cane plants, costs the sugarcane industry a billion US dollars in crop damage and processing costs yearly. The propagation of the first biotechnology-derived Cry1Ab-expressing sugarcane variety was approved and launched in Brazil in late 2017. Cry1Ab and Cry1Ac proteins target receptors found only in lepidoptera, causing selective toxicity. These proteins have proved very effective, as their toxicity is specific, and can thus be used to target specific lepidoptera pests. Given these attributes, research has shown that Cry1Ab and Cry1Ac are useful in sugarcane agronomy and production, benefits including improved plant protection and reduced pesticide use. The Brazilian sugarcane processing industry is highly integrated and focused on the production of ethanol (for primarily domestic energy markets) and sugar for domestic and export markets. Careful analysis of the foreign trade information regarding Brazilian sugarcane-derived products exported to key markets show that the article of commerce relating to human food is sugar, either raw sugar, or refined sugar. The by-products of sugarcane processing, such as the bagasse (fiber) and molasses are recycled within industrial processing employed by Brazilian mills and are not exported in any appreciable amounts globally. This trade situation, and the highly refined nature of either raw or refined sugar, creates the possibility to consider a broad-based approach to establishing the safety of many widely-used proteins based on sound scientific and policy foundation. A key aspect of sugarcane processing and the production of raw and refined sugar from sugarcane, involves the extensive processing with heat and pH adjustment that effectively removes all detectable DNA and proteins from raw and refined sugars (Cheavegatti-Gianotto et al., 2011).

Various sugarcane processing studies done by Cullis et al. (2014) who examined total DNA and protein loss, by Cheavegatti-Gianotto et al. (2018) who studied Rubisco and Cry1Ab loss in Bt sugarcane and by Joyce et al. (2013) have established that sugarcane processing effectively eliminates detectable DNA and protein from raw or refined sugar. Other studies measuring specific transgene DNA or protein in sugar beets have also shown that raw or refined sugar produced from sugar beets do not contain detectable DNA or protein. In a comprehensive study, Cullis et al. (2014) established that total protein levels were below 1 microgram per-gram of refined sugar; however, the theoretical levels of newly-expressed GM protein would be orders of magnitude below this value, depending ultimately on the level of protein expression as a percent of total protein content. Since the presence of DNA and protein in raw and refined sugar examined by Cullis et al. (2014) and Cheavegatti-Gianotto et al. (2018) were below the limit of quantification, even using highly sensitive methods, any possible human dietary exposure to these proteins would be extremely low.

In this study, the safety of four commonly used proteins in GM crops were examined; the Cry1Ab and Cry1Ac proteins used in crops to control lepidoptera pests, NPTII, a commonly used selectable marker protein and Bar, an herbicide-tolerance trait also used as a selectable marker. These proteins have been studied extensively and approved widely by regulatory agencies worldwide. Results of acute toxicology studies have established No Observed Adverse Effect Levels (NOAELs) and significant confirmatory data exists from a variety of subchronic toxicity and other studies. Digestibility studies *in vitro* have shown that these proteins are rapidly degraded in either mock gastric or intestinal fluids indicating that they are digested readily and not available for oral absorption. Using this wealth of information, it is possible to evaluate the safety of sugarcane products expressing these proteins using a first-principles approach that incorporated the extremely low theoretical amounts in raw or refined sugar, the known intakes of added sugar in various representative export markets and the established safety of these proteins. For these four proteins, the country specific added sugar average intake values were examined using two scenarios of possible presence in raw/refined sugar. This exposure-driven approach established extremely large safety margins for these proteins and provides a general approach to safety assessment of other highly processed food and feed ingredient products derived from GM crops.

## Methods

### Estimation of the theoretical concentration of the newly expressed protein in raw and refined sugar: worst-case and reasonable-case assumptions

Using the results of Cullis et al. (2014) and Cheavegatti-Gianotto et al. (2018) and Reasonable assumptions regarding the level of expression of Cry1Ac, Cry1Ab, NPTII, and Bar proteins in stalk, two scenarios were developed regarding possible presence of these proteins in raw or refined sugar. It is important to note that the varieties of sugarcane that may contain the four proteins examined are/will be developed for cultivation in Brazil (Center-South and Northeast) and will not be commercialized elsewhere. Therefore, the potential exposure of consumers outside of Brazil will occur via the processing and export of either raw or refined sugar.

The first Scenario assumes that the concentration estimate of each of the four newly-expressed proteins is 1 μg newly-expressed protein/g refined sugar. This “Worst-case” estimate clearly is a significant overestimate of the actual concentration of each newly-expressed protein/g raw or refined sugar. Cullis et al. (2014) examined the loss of TOTAL sugarcane stalk protein during the processing of sugar in Brazil. Using sensitive detection methods, they found that protein was not detectable using these methods at < 1 μg/g refined sugar. Consequently, Scenario A is based on this detection level and assumes that all the protein in the sugar is that specific protein (e.g., Cry1Ab). The second scenario, described herein as “Reasonable-case,” assumes that the concentration of the specific newly-expressed transgenic protein represent 0.1% of total stalk protein, a more Reasonable scenario given the experiences to date with GM sugarcane. Therefore, for the “Reasonable-case” scenario, it is assumed that the actual concentrations of the newly-expressed proteins are only 0.1% of the 1 μg/g refined sugar value reported by Cullis et al. (2014) reported for total protein. In the case of CTC's Bt sugarcane recently approved in Brazil, the actual expression levels of both Cry1Ab and NPTII were much lower than 0.1% of total stalk protein, both of which were below the limit of quantification of 235 and 34 ng/g stalk tissue, respectively (Cheavegatti-Gianotto et al., 2018).

In conclusion, the assumed concentration of newly-expressed protein in refined sugar was 1 ppm for the “Worst-case” and 1 ppb for the “Reasonable-case” scenarios. It is noteworthy that concentrations in sugar consumed chronically in the various eight country markets will be diluted substantially by non-Brazilian sourced raw or refined sugar; for example, the contribution of imported sugar from Brazil to the amount of added sugar consumed in the Canada and the US is estimated at 11% (FAO/WHO, 2000).

### Estimation of added sugar mean intakes and derived “worst-case” and “reasonable-case” intakes of newly expressed proteins in eight representative markets of exported brazilian raw and refined sugar

It is not simple to compare similar data regarding added sugar consumption across various countries as differing methods are used in government-funded surveys of food intakes and composition. In our study, data collected in 2015 by Euromonitor International was used (Ferdman, 2015). Euromonitor International nutrition methodologies assess the probable mean intakes/person/day of eight nutrients: energy, protein, carbohydrates, sugar, fat, saturated fat, fiber and salt. The examined packaged foods and fresh foods and beverages based on nutrient content information and intake information in 54 countries globally. This study approach using consistent market research methodologies was used in our case study because it represented a similar approach in all countries examined herein.

The mean sugar intakes reported by Euromonitor International across various countries were typically higher than those found in published research. For example, research performed by the Canadian Sugar Institute, found average sugar intake to be 50 (g/p/d) compared to the 89.1(g/p/d) value found by Euromonitor International marketing research. Marketing research values were also higher than the USDA NHANES values in the United States, the former being 126.4 (g/p/d) compared with the NHANES data of 82 (g/p/d). The differences in intake survey-based results, like those described by the Canadian Sugar Institute and USDA NHANES, compared with the Euromonitor International results, are likely due to differences in methodology. The Euromonitor International data reports food disappearance vs. food consumption as estimated by dietary surveys. As a result, the Euromonitor International results shown in Tables 4, 5 are likely overestimates of actual ingredient intake. Therefore, for consistency across geographies, these Euromonitor International “overestimate” ingredient intake results were preferred and used in both our “worst case” and “reasonable case” estimates of sugar intake.

All estimated “Worst-case” and “Reasonable-case” estimates of theoretical protein intake for the four subject Cry1Ab, Cry1Ac, NPTII, and Bar proteins were calculated using the Euromonitor International marketing research mean added sugar values and assumed concentrations in sugar of 1 ppm and 1 ppb, respectively. Using these estimated intakes (Tables 4, 5), and the internationally accepted NOAELs for the Cry1Ab, Cry1Ac, NPTII, and Bar proteins, it was possible to calculate safety margins as the NOAEL for each protein divided by estimated average protein intakes from added sugar in the eight countries. In order to evaluate the safety of these proteins, it is necessary to summarize the toxicology and related safety data for these individual four proteins.

### Summary of toxicology and safety information on the four newly-expressed proteins in GM sugarcane: published literature and NOAEL values

The four specific proteins selected in this assessment were chosen because both the Cry1Ab and Cry1Ac proteins are useful in the management of sugarcane borer in Brazilian sugarcane production and the NPTII and Bar proteins are widely used as selectable markers. Each of these proteins have both been widely used in other agricultural biotechnology crops and extensively studied and reviewed by regulatory agencies worldwide. As shown in Table 2, the US Environmental Protection Agency (EPA) has reviewed the safety of these pesticidal (Cry1Ab and Cry1Ac) and inert (NPTII and Bar) proteins. EPA concluded, based on a variety of the data presented below, that these proteins were safe for their potential use in all crops, including possible use in sugarcane. This conclusion was based on considerations like history of safe use (in bacterial pesticidal sprays used in organic agriculture), animal toxicology studies, studies on the digestibility of the proteins and bioinformatics studies for potential allergens or toxins. The safety assessments conclusions referenced in this manuscript were written by EPA and European Food Safety Authority (EFSA) and based on published literature and product-specific submissions by Bayer, Monsanto, Syngenta and other companies. The studies and assessments follow the Codex Alimentarius “Guideline for the Conduct of Food Safety Assessment of Foods Derived from Recombinant-DNA Plants” (2003). These assessments included the following information described below: acute and subchronic toxicology studies, *in vitro* digestibility and heat lability studies, and bioinformatics assessments of potential allergenicity and toxigenicity. The key results for each newly-expressed protein are summarized below that led to the determination of the individual NOAEL for each protein.

### Acute toxicology studies

Table 1 shows the results of Acute and Subchronic toxicology studies for Cry1Ac, Cry1Ab, NPTII, and Bar. The studies were primarily conducted using mice administered the protein either by acute gavage or in the diet; the exceptions being that an acute toxicity study on Bar protein was done using intravenous dosing and a subchronic study that was conducted in mice. The goal of these studies was to determine the NOAEL for the specific tested substances. There were no adverse effects of any protein at the highest dose tested and therefore the highest dose tested is the NOAEL. Often the highest dose tested was due to physical chemical constraints like the solubility of the protein in the injection solution. The results (Table 1) shows that the oral NOAEL for Cry1Ab and Cry1Ac were ≥ 4,000 and 1,460 mg/kg bw, respectively. Similarly, the oral NOAEL for NPTII was found to be ≥ 5,000 mg/kg bw. The highest dose tested was either limited by the solubility of the protein in the dosage formulation or the accepted maximum dose tested in 5,000 mg/kg bw acute toxicity limit test. These results establish that these proteins are essentially non-toxic. By comparison to the studies with Cry1Ab, Cry1Ac, and NPTII proteins, the NOAEL of the Bar protein was determined following intravenous dosing and not oral dosing. There were no effects observed after 10 mg/kg bw iv dosing, the highest dose administered. Given that the Bar protein was rapidly degraded in simulated gastric and intestinal juices incubations (within seconds to minutes), it is reasonable to expect that the NOAEL for Bar protein orally is several orders of magnitude higher than the iv NOAEL dosing value of 10 mg/kg bw. Regardless, as a result of this difference in route of administration and dosing limitation, the NOAEL for Bar is the lowest amongst these proteins.

**Table 1:** Toxicology study results for Cry1Ab, Cry1Ac, NPTII, and bar proteins.

	**Cry1Ab**	**Cry1Ac**	**NPTII**	**Bar**
Acute Toxicology	Protein was orally administered to mice. Five male and five female mice were given doses up to 4,000 mg/kg bw. No effects were observed.	Protein was orally administered to 8–10 week mice. Mice were given a single dose at 1,280–1,290 mg/kg bw. No effects were observed.	Protein was orally administered to 10 males and 10 female mice. Doses were administered 100, 1,000, and 5,000 mg/kg bw. No effects were observed.	Protein was intravenously administered to mice at doses of 1 and 10 mg/kg bw. No effects were observed.
Subchronic Toxicology	Rice containing Cry1Ab was feed to mice over a 13 week period. Rodents were fed rice at three separate protein concentrations (17.5, 35, and 70 %). No effects were observed.	Cry1Ac containing diets were administered to mice over a 13 weeks period. Rodents were fed maize at three separate protein concentrations (12.5, 25, 50%). No detectable Cry1Ac-M protein was found in the serum of rats after feeding diets containing GM maize for 3 months. The results demonstrated that BT-38 maize is as safe as conventional non-GM maize.	NPTII containing diets were administered to mice over a 13 weeks period. Rodents were fed maize at three separate protein concentrations (12.5, 25, 50%). No detectable NPTII protein was found in the serum of rats after feeding diets containing GM maize for 3 months. No effects were observed. Studies done on BT-38 maize.	Protein was incorporated in the diet of mice for 90 days at levels of 0, 5, and 50 g/kg diet, this was done over a 2 week period. Corresponding to 7.6 and 7.9 mg/kg BW/day for males and females. No effects were observed.
NOAEL (mg/kg bw)	>4,000	>1,460	>5,000	>10

### Subchronic toxicology

Along with acute toxicology studies, focused on finding the NOAELs for Cry1Ac, Cry1Ab, NPTII, and Bar, subchronic toxicology studies were also performed in an effort to study possible longer-term effects of the proteins. In these studies, the proteins were administered orally, either by gavage or in the diet (as a constituent of GM grain used to formulate the diets), for 90 days. As Table 1 shows no adverse effects observed at the highest doses administered for these proteins. In addition to the results of these studies, numerous other studies have been conducted and report on the grain/processed fraction produced from Cry1Ab and Cry1Ac-expressing biotechnology crops. These studies confirmed that there were no adverse effects at the highest doses tested. As a consequence of the entire weight of the evidence regarding the safety of these protein, toxicologists and regulators have concluded that the most appropriate NOAELs are those determined by the acute toxicology studies noted above.

### *In vitro* digestion stability tests

Digestive stability testing was performed in a mock *in vitro* digestive environment simulating both a gastric and intestinal fluid. The goal of these tests is to estimate the rate of protein degradation or denaturation in the human digestive systems. Proteins that are rapidly denatured (by low pH) and enzymatically digested (by intestinal enzymes) have a lower probability of producing either toxicity since digestion products are common peptides or dietary amino acids or allergenicity because the protein is not present to elicit antibody production or allergic reactions. Cry1Ab, Cry1Ac, NPTII, and Bar are all degraded quickly in these *in vitro* mock digestive solutions, making them significantly less likely to exhibit local or systemic toxicity or allergenicity, as protein exposure is transient. The conclusions from the results of these tests, as concluded by EPA, for the Cry1Ab, Cry1Ac, NPTII, and Bar proteins, are provided below.

#### Cry1Ab

“The *in vitro* digestion assays confirm that the protein is being broken down in the presence of typical digestive fluids and is not unusually persistent in the digestive system. All were degraded in gastric fluid in 0–7 min” (Kough et al., 2010).

#### Cry1Ac

“The Cry1Ac protein was digested within 30 s in simulated gastric fluid containing pepsin. Small peptides remaining following gastric simulated digestion were completely degraded to amino acid residues in SIF (simulated intestinal fluid) upon contact” (Kough et al., 2010).

#### NPTII

“NPTII degrades extremely readily in SGF (simulated gastric fluid). No NPTII protein was detected, by western blot analysis, at the first incubation time point of 10 s. In SIF (simulated intestinal fluid), NPTII also degrades readily with 50% degradation occurring after 2–5 min of incubation at 37°C.” (Fuchs et al., 1993)

#### Bar

“Bar proteins were degraded very rapidly and completely in the SGF (simulated gastric fluid) (pH 2) or SIF (simulated intestinal fluid) (pH 7.5), within few seconds of incubation, in the presence of pepsin or pancreatin, respectively. … In the SIF (simulated intestinal fluid) assay, the complete degradation of remaining 7-kDa fragments was achieved within 5 min rather than a few seconds.” (Hérouet et al., 2004).

### EPA exemptions from tolerance

After having reviewed the extensive database supporting the safety of these four proteins separately, EPA concluded that the proteins have safety profiles that permit them to be exempted from the need for tolerances in food or feed in the United States. The EPA issued these exemptions from tolerances for the Cry1Ab, Cry1Ac, NPTII, and Bar proteins for use in any crop based on several factors including the very high NOAELs. These EPA exemptions are listed below in Table 2.

**Table 2:** EPA exemptions from tolerances for Cry1Ab, Cry1Ac, NPTII, and Bar.

**Cry1Ab (*Bacillus thuringiensis*)**	**Cry1Ac (*Bacillus thuringiensis*)**	**Bar (Phosphinothricin Acetyltransferase (PAT) enzyme)**	**NPTII (neomycin phosphotransferase II)**
“Residues of *Bacillus thuringiensis* Cry1Ab protein in all plants are exempt from the requirement of a tolerance when used as plant-incorporated protectants in all food commodities.” → **40 CFR 174. 511**	“Residues of *Bacillus thuringiensis* Cry1Ac protein in all plants are exempt from the requirement of a tolerance when used as plant-incorporated protectants in all food commodities.” → **40 CFR 174.510**	“Residues of the Phosphinothricin Acetyltransferase (PAT) enzyme are exempt from the requirement of a tolerance when used as plant-incorporated protectant inert ingredients in all food commodities.” → **40 CFR 174.522**	“Residues of the neomycin phosphotransferase II (NPTII) enzyme are exempted from the requirement of a tolerance in all food commodities when used as a plant-incorporated protectant inert ingredient.” → **40 CFR 174.521**

### International approvals for the newly-expressed proteins

The safety of these proteins has also been widely reviewed by regulatory agencies globally. As shown in Table 3, products containing the newly-expressed proteins Cry1Ac, Cry1Ab, NPTII, and Bar have been approved for consumption in many countries worldwide. Given the breadth of biotechnology crops utilizing these proteins, these approvals further confirm the conclusions drawn by the US EPA and the European Union EFSA regarding the safety of the food and feed produced from these crops.

**Table 3:** Country and product approvals for Cry1Ab, Cry1Ab, NPTII, and bar.

**Protein**	**Number of countries to approve**	**Number of events approved**	**Crops approved in**
Cry1Ac	54	38	Cotton, Eggplant, Maize, Poplar, Rice, Soybean, Tomato, Sugarcane
Cry1Ab	55	57	Alfalfa, Apple, Canola, Chicory, Cotton, Eggplant, Eucalyptus, Flax, Maize, Melon, Papaya, Plum, Popular, Potato, Squash, Sugar beet, Sugarcane, Sugar Beet, Tobacco, Tomato
NPTII	57	121	Alfalfa, Apple, Canola, Chicory, Cotton, Eggplant, Eucalyptus, Flax, Maize, Melon, Papaya, Plum, Popular, Potato, Squash, Sugar beet, Sugarcane, Sugar Beet, Tobacco, Tomato
Bar	47	55	Canola, Chicory, Cotton, Maize, Rice, Soybean, Sugarcane

### Calculated safety margins

Based on the “Worst-case” and “Reasonable-case” theoretical protein exposure values from added sugar and the NOAEL values for each of the four proteins, safety margins for the Cry1Ab, Cry1Ac, NPTII, and Bar proteins were calculated. These safety margin values were calculated by dividing the protein-specific NOAELs, expressed in μg/kg bw/d, by the mean exposure estimate also expressed as μg/kg bw/day.

## Results

The mean added sugar intakes in the eight selected countries that are markets for Brazilian-produced sugar varied significantly, probably as a result of dietary preferences and socio-economic factors. Calculated safety margins for the four newly-expressed proteins for the “Worst-case” and “Reasonable-case” exposure scenarios are provided in Tables 4, 5, respectively. Mean added sugar consumptions for the eight sample countries are also shown in Tables 4, 5. The tables show India, China and Indonesia to be the low consumers, Russia and Brazil to be intermediate consumers, and Japan, Canada and the United States to be the higher consumers. It appears that dietary preferences and socio-economic differences between the countries may contribute this broad distribution in average added sugar intakes. Regardless, the theoretical intakes at the “Worst-case” and “Reasonable-case” scenarios are related directly with the sugar intake figures: theoretical protein intakes were lower in India, China and Indonesia, intermediate in Russia and Brazil, and highest in Japan, Canada and the United States.

**Table 4:** Safety margins at “Worst-case” Protein exposure in eight countries.

**Country**	**Marketing research mean added sugar consumption (g/p/d)**	**Worst-case sugar intake (mg/kg bw/d)**	**Maximum protein exposure (μg/kg bw/d)**	**Calculated safety margins Cry1Ab**	**Calculated safety margins Cry1Ac**	**Calculated safety margins NPTII**	**Calculated safety margins bar**
Brazil (a)	47.6	0.79	0.79	5.1 × 10^6^	1.8 × 10^6^	6.3 × 10^6^	1.3 × 10^4^
Canada (b)	89.1	1.49	1.49	2.7 × 10^6^	9.8 × 10^5^	3.4 × 10^6^	6.7 × 10^4^
China (c)	15.7	0.26	0.26	1.5 × 10^7^	5.6 × 10^6^	1.9 × 10^7^	3.8 × 10^4^
Indonesia (d)	15.2	0.25	0.25	1.6 × 10^7^	5.8 × 10^6^	2.0 × 10^7^	4.0 × 10^4^
India (e)	5.1	0.085	0.085	4.7 × 10^7^	1.7 × 10^7^	5.9 × 10^7^	1.2 × 10^5^
Japan (f)	56.7	0.95	0.95	4.2 × 10^6^	1.5 × 10^6^	5.3 × 10^6^	1.1 × 10^4^
Russia (g)	20.0	0.33	0.33	1.2 × 10^7^	4.4 × 10^6^	1.5 × 10^7^	3.0 × 10^4^
US (h)	126.4	2.11	2.11	1.9 × 10^6^	6.9 × 10^5^	2.4 × 10^6^	4.7 × 10^3^

**Table 5:** Safety margins at “Reasonable-case” protein exposure in eight countries.

**Country**	**Marketing research sugar consumption (g/p/d)**	**Worst-case sugar intake sugar consumption (mg/kg bw/d)**	**Reasonable protein theoretical exposure (μg/kg bw/d)**	**Calculated safety margins Cry1Ab**	**Calculated safety margins Cry1Ac**	**Calculated safety margins NPTII**	**Calculated safety margins bar**
Brazil (a)	47.6	0.79	0.00079	5.1 × 10^9^	1.8 × 10^9^	6.3 × 10^9^	1.3 × 10^7^
Canada (b)	89.1	1.49	0.00149	2.7 × 10^9^	9.8 × 10^8^	3.4 × 10^9^	6.7 × 10^7^
China (c)	15.7	0.26	0.00026	1.5 × 10^10^	5.6 × 10^9^	1.9 × 10^10^	3.8 × 10^7^
Indonesia (d)	15.2	0.25	0.00025	1.6 × 10^10^	5.8 × 10^9^	2.0 × 10^10^	4.0 × 10^7^
India (e)	5.1	0.085	0.000085	4.7 × 10^10^	1.7 × 10^10^	5.9 × 10^10^	1.2 × 10^8^
Japan (f)	56.7	0.95	0.00095	4.2 × 10^9^	1.5 × 10^9^	5.3 × 10^9^	1.1 × 10^7^
Russia (g)	20.0	0.33	0.00033	1.2 × 10^10^	4.4 × 10^9^	1.5 × 10^10^	3.0 × 10^7^
US (h)	126.4	2.11	0.00211	1.9 × 10^9^	6.9 × 10^8^	2.4 × 10^9^	4.7 × 10^6^

Table 4 also shows the calculated safety margins for each newly-expressed protein using the “Worst-case” scenario exposure in the eight selected countries. The “Worst-case” scenario safety margins in the eight countries for the three proteins for which the NOAELs were established by oral dosing (i.e., Cry1Ab, Cry1Ac, and NPTII) ranged from 6.9 × 10^5^ to 4.7 × 10^7^; the safety margins for the Bar protein were lower as a result of the lower NOAEL value at the highest dose tested following intravenous dosing. The resulting Bar protein safety margins were lower and ranged from 4.7 × 10^3^ to 1.2 × 10^5^. The lower safety margins for the Bar protein was based on the fact that the protein was administered intravenously at 10 mg/kg bw; the actual oral NOAEL for Bar would undoubtedly be orders of magnitude higher given the rapid degradation in both simulated gastric and intestinal fluids. Nonetheless, the safety margins for all four proteins, using the “Worst-case” scenario, were at least 10^3^, well above those considered by toxicologists and regulators globally to establish dietary safety.

Table 5 shows the calculated safety margins for each newly-expressed protein using the “Reasonable-case” scenario in the eight selected countries. The “Reasonable-case” scenario safety margins in the eight countries for the three proteins for which the NOAELS were established by oral dosing (i.e., Cry1Ab, Cry1Ac, and NPTII) ranged from 6.9 × 10^8^ to 4.7 × 10^10^; the safety margins for the Bar protein were lower and ranged from 4.7 × 10^6^ to 1.2 × 10^8^. The safety margins for the “Reasonable-case” scenario, were at least 10^6^, well above those considered by toxicologists and regulators globally to establish the safety.

## Discussion

CTNBio, the Brazilian government regulatory authority involved in the review and approval of biotechnology-derived products, recently approved a Cry1Ab-expressing sugarcane plant for cultivation in Brazil (CTNBio, 2017). The CTNBio assessment considered a wide range of data on the Cry1Ab-expressing sugarcane variety including agronomic and phenotypic studies, non-target organism studies, molecular and protein characterization, protein expression in sugarcane tissues, effects of sugarcane processing on DNA and protein in raw and refined sugar, and product food and feed safety assessment. Based on these assessments, CTNBio approved the product for cultivation in the Center-South growing region in Brazil. With this approval, CTC has started controlled bulk-up/propagation field activities and commercial scale sugar production will occur in 2020.

Brazil is a major supplier of raw or refined sugar globally. Analysis of the export data over the last 5 years of sugarcane-derived products from Brazil show that the vast majority of exported sugarcane-derived products is raw or refined sugar; consequently, the major articles of commerce are highly purified ingredients. Brazilian foreign trade exports to the top 20 sugar markets, which include most of the eight countries studied herein, shows that virtually all of sugarcane-derived Brazilian exports are raw or refined sugar. A trace amount of exports of distilled alcoholic beverages does occur in some countries but the fermentation and distillation process would certainly remove proteins. The exported raw sugar is at least 97% pure sucrose (OECD, 2011). In many countries, food regulations require that raw sugar for human consumption be further refined, to avoid contamination which may occurs during transport of commodities, and the final purity of refined sucrose is over 99.7 % (OECD, 2011).

As a result of the high temperatures, pH adjustments and sucrose crystallization conditions produced in the processing of sugarcane stalk to raw and refined sugar it is not surprising that sugar does not contain detectable quantities of DNA or protein. Several investigators have evaluated this processing loss of DNA and protein, examining the loss of both endogenous and exogenous DNA or protein including total protein and DNA and Rubisco DNA and protein (Cullis et al., 2014; Cheavegatti-Gianotto et al., 2018). This conclusion is further substantiated by Cheavegatti-Gianotto et al. (2018) including data on the lack of detection of Cry1Ab in processing fractions including clarified juice, raw and refined sugar produced from Bt sugarcane in Brazil. Similar findings have been reported for loss of newly-expressed proteins in GM sugarcane (Joyce et al., 2013) and sugar beets (Klein et al., 1998; Oguchi et al., 2009).

The highly purified nature of raw and refined sugar allowed for the development of a generalized safety assessment Case Study given the low detection limits and range of sugar consumption levels worldwide. The seven export countries researched in this studied, in addition to Brazil itself, were chosen because they are chief importers of Brazilian sugar and, more importantly, because they all have established regulatory agencies that review biotechnology-derived crops and derived food ingredients. The Cry proteins researched in this study, both of which have been proven extremely effective against sugarcane borer, were chosen as they have been widely approved around the world including in the eight countries researched in this study. The selectable marker proteins researched, NPTII and Bar, were chosen as they were also approved in all eight of the sample countries.

Based on the marketing research data conducted by EuroMonitor International, we were capable of examining mean added sugar intake in the eight sample countries using similar methodologies. Several of these countries conduct nutritional intake surveys and analyses to examine nutritional trends and develop nutritional guidance; however, across-country comparisons, often using differing methodologies, are not amenable to side-by-side comparisons. Therefore, the marketing research approach used by Euromonitor International was used. The results of mean sugar intake data showed a large range with the lowest intakes occurring in developing economies (India, Indonesia, China and Brazil) and the higher intakes in more developed economies (United States, Canada and Japan).

The proteins examined in this study have been widely used in agricultural biotechnology products globally. The Cry1Ab and Cry1Ac proteins have been shown to be effective in managing lepidoptera pests in various crops including in sugarcane production in Brazil. The NOAELs, the highest dose tested that was not associated with any adverse effects in animals for these four proteins, are generally well established and accepted by regulatory agencies including the US EPA and the European Union EFSA. Safety margin results for Cry1Ab and Cry1Ac were very high for both scenarios. For Cry1Ac the “Worst-case” safety margins ranged from 6.9 × 10^5^ to 1.7 × 10^7^, while the “Reasonable-case” safety margins ranged from 6.9 × 10^8^ to 1.7 × 10^10^ (see Table 4). For Cry1Ab the “Worst-case” safety margins ranged from 1.6 × 10^6^ to 4.7 × 10^7^, while the “Reasonable-case” safety margins ranged from 1.6 × 10^9^ to 4.7 × 10^10^ (see Table 5). The immensity of the safety margins presented here are difficult to interpret in the abstract. Typically, safety margins of 100–500 is required for a new food ingredient added to food or beverage; in other words, the allowable daily intake of the ingredient is often determined as the lowest NOAEL divided by 100 (i.e., a 100 fold safety margin). The safety margins calculated under the “Worst-case” and “Reasonable-case” exposures for sugar are several orders of magnitude higher than those used by food toxicologists and regulators for other food ingredients. It is also possible to consider the extremely large safety margins for sugar in the context of the amount of sugar that would need to be consumed to reach the NOAEL values. For example, using Cry1Ac protein safety (1,460 mg/kg bw), a 60 kg person consuming refined sugar with a Cry1Ac concentration < 1 μg/g sugar would need to ingest 87.6 metric tons or 192,720 lbs of added sugar to theoretically exceed the NOAEL value.

The scenarios studied were chosen because they represent extreme overestimates. The “Worst-case” scenario assumed that all of the possible protein present at the Cullis et al. (2014) limit of quantification was the protein of interest—clearly a great overestimation. Even the “Reasonable-case” scenario assumed that 0.1% of the total stalk protein was the protein of interest. In addition, these scenarios do not take into account two important sources of “dilution” of the raw and refined sugar produced from Brazilian biotechnology-derived varieties; the first is dilution within Brazil by non-GM derived sugar and the second is dilution in the local market (e.g., India, Japan, US). Brazilian varieties expressing Cry1Ab or Cry1Ac will be suited for specific growing regions. Given that plant propagation of new varieties is slow, taking 3 years to reach only one-two percent market share, it is reasonable that the proportion of Brazilian sugarcane that is biotechnology-derived and expressing Cry1Ab or Cry1Ac will be less than 20% for several years to come. The dilution of Brazilian produced sugar by other sources of sugar also significantly lowers the possible protein concentrations studied in the various scenarios and export countries. For example, in the case of the United States, only 1–2% of sugar consumed by Americans is produced in Brazil. This means, at least for the United States, the safety factor could be up to another two orders of magnitude higher. Again, the purpose of using the “Worst-case” and “Reasonable-case” scenarios were to provide tangible examples of the relationship between exposures and safety margins, especially for such highly purified ingredients. The overall conclusion of this study is that the possible exposures to these four newly expressed proteins are trivial compared with the known safety NOAELs for these proteins. These conclusions are valid regardless of the exact level of protein expression in the stalk. It is, of course, necessary to confirm, on a variety-by-variety basis, that the expressed proteins are identical to the proteins tested for the determination of NOAELs. Given the lack of detection of protein at low limits of detection, it should be possible to regulators worldwide to consider a significantly reduced data package to support the import of highly purified raw and refined sugar.

Finally, the general approach used in this study could be expanded to include other processed food ingredients derived from GM plants, including products like oils, oil fractions, lecithin and vitamins, where the processes are well established and the quantity of newly-expressed proteins are present consistently at *very low concentrations*.

## Author contributions

JH and RL: conceived the study; JH and RK: analyzed the data; RK, JH, and AC-G: wrote the manuscript; AC-G and WdO: provided supportive information.

### Conflict of interest statement

The authors of this publication, RK, RL and JH are consultants to CTC on biosafety studies of products related to the research being reported. The authors of this publication, AC and WdO, are employees of CTC which is developing products related to the research being reported.
